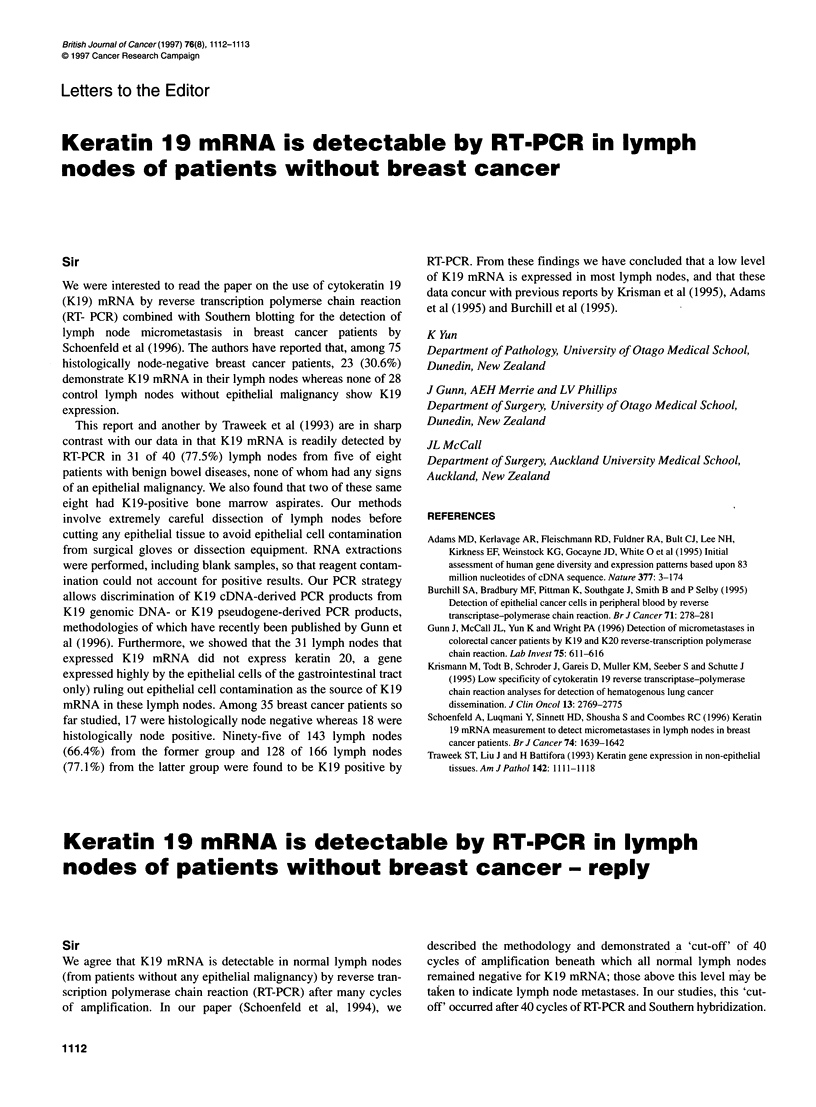# Keratin 19 mRNA is detectable by RT-PCR in lymph nodes of patients without breast cancer.

**DOI:** 10.1038/bjc.1997.516

**Published:** 1997

**Authors:** K. Yun, J. Gunn, A. E. Merrie, L. V. Phillips, J. L. McCall


British Journal of Cancer (1997) 76(8), 1112–1113
© 1997 Cancer Research Campaign

Letters to the Editor

### Keratin 19 mRNA is detectable by RT-PCR in lymph nodes of patients without breast cancer

**Sir**

We were interested to read the paper on the use of cytokeratin 19 (K19) mRNA by reverse transcription polymerse chain reaction (RT- PCR) combined with Southern blotting for the detection of lymph node micrometastasis in breast cancer patients by Schoenfeld et al (1996). The authors have reported that, among 75 histologically node-negative breast cancer patients, 23 (30.6%) demonstrate K19 mRNA in their lymph nodes whereas none of 28 control lymph nodes without epithelial malignancy show K19 expression.

This report and another by Traweek et al (1993) are in sharp contrast with our data in that K19 mRNA is readily detected by RT-PCR in 31 of 40 (77.5%) lymph nodes from five of eight patients with benign bowel diseases, none of whom had any signs of an epithelial malignancy. We also found that two of these same eight had K19-positive bone marrow aspirates. Our methods involve extremely careful dissection of lymph nodes before cutting any epithelial tissue to avoid epithelial cell contamination from surgical gloves or dissection equipment. RNA extractions were performed, including blank samples, so that reagent contamination could not account for positive results. Our PCR strategy allows discrimination of K19 cDNA-derived PCR products from K19 genomic DNA- or K19 pseudogene-derived PCR products, methodologies of which have recently been published by Gunn et al (1996). Furthermore, we showed that the 31 lymph nodes that expressed K19 mRNA did not express keratin 20, a gene expressed highly by the epithelial cells of the gastrointestinal tract only) ruling out epithelial cell contamination as the source of K19 mRNA in these lymph nodes. Among 35 breast cancer patients so far studied, 17 were histologically node negative whereas 18 were histologically node positive. Ninety-five of 143 lymph nodes (66.4%) from the former group and 128 of 166 lymph nodes (77.1%) from the latter group were found to be K19 positive by RT-PCR. From these findings we have concluded that a low level of K19 mRNA is expressed in most lymph nodes, and that these data concur with previous reports by Krisman et al (1995), Adams et al (1995) and Burchill et al (1995).

*K Yun*
*Department of Pathology, University of Otago Medical School, Dunedin, New Zealand*

*J Gunn, AEH Merrie and LV Phillips*
*Department of Surgery, University of Otago Medical School, Dunedin, New Zealand*

*JL McCall*
*Department of Surgery, Auckland University Medical School, Auckland, New Zealand*

**REFERENCES**

Adams MD, Kerlavage AR, Fleischmann RD, Fuldner RA, Bult CJ, Lee NH, Kirkness EF, Weinstock KG, Gocayne JD, White O et al (1995) Initial assessment of human gene diversity and expression patterns based upon 83 million nucleotides of cDNA sequence. *Nature* **377**: 3–174

Burchill SA, Bradbury MF, Pittman K, Southgate J, Smith B and P Selby (1995) Detection of epithelial cancer cells in peripheral blood by reverse transcriptase–polymerase chain reaction. *Br J Cancer* **71**: 278–281

Gunn J, McCall JL, Yun K and Wright PA (1996) Detection of micrometastases in colorectal cancer patients by K19 and K20 reverse-transcription polymerase chain reaction. *Lab Invest* **75**: 611–616

Krismann M, Todt B, Schroder J, Gareis D, Muller KM, Seeber S and Schutte J (1995) Low specificity of cytokeratin 19 reverse transcriptase–polymerase chain reaction analyses for detection of hematogenous lung cancer dissemination. *J Clin Oncol* **13**: 2769–2775

Schoenfeld A, Luqmani Y, Sinnett HD, Shousha S and Coombes RC (1996) Keratin 19 mRNA measurement to detect micrometastases in lymph nodes in breast cancer patients. *Br J Cancer* **74**: 1639–1642

Traweek ST, Liu J and H Battifora (1993) Keratin gene expression in non-epithelial tissues. *Am J Pathol* **142**: 1111–1118